# A 3D Computational Model of Transcutaneous Electrical Nerve Stimulation for Estimating Aβ Tactile Nerve Fiber Excitability

**DOI:** 10.3389/fnins.2017.00250

**Published:** 2017-05-16

**Authors:** Kaihua Zhu, Liming Li, Xuyong Wei, Xiaohong Sui

**Affiliations:** School of Biomedical Engineering, Shanghai Jiao Tong UniversityShanghai, China

**Keywords:** 3D computational modeling, tactile sensory feedback, transcutaneous electrical nerve stimulation, Aβ tactile nerve fiber, double-cable model, psychophysical experiments

## Abstract

Tactile sensory feedback plays an important role in our daily life. Transcutaneous electrical nerve stimulation (TENS) is widely accepted to produce artificial tactile sensation. To explore the underlying mechanism of tactile sensation under TENS, this paper presented a novel 3D TENS computational model including an active Aβ tactile nerve fiber (TNF) model and a forearm finite element model with the fine-layered skin structure. The conduction velocity vs. fiber diameter and strength-duration relationships in this combined TENS model matched well with experimental data. Based on this validated TENS model, threshold current variation were further investigated under different stimulating electrode sizes with varied fiber diameters. The computational results showed that the threshold current intensity increased with electrode size, and larger nerve fibers were recruited at lower current intensities. These results were comparable to our psychophysical experimental data from six healthy subjects. This novel 3D TENS model would further guide the floorplan of the surface electrodes, and the stimulating paradigms for tactile sensory feedback.

## Introduction

Tactile sense is important for us to gather information about the world, and tactile sensory feedback plays a great role in our daily life. Both tactile sensation and motor function are important for reali zing dexterous manipulation. In recent years, incorporation of artificial tactile sensory feedback has obtained great interest worldwide to help achieve the fine closed-loop control for the prosthetic hand (Kim et al., 2014; Tan et al., 2014; Tee et al., 2015).

For stroke patients with severe loss of hand sensation, their grasp function could be greatly improved by incorporation of the fingers' artificial tactile sensation based on mechanical stimulation of other normal skin area such as shoulder (Kita et al., 2013). By combining both tactile and visual feedback, the virtual reality-based training technology made a significant role in rehabilitation for patients with stroke or Parkinson disease (Saposnik et al., 2010; Liao et al., 2014). For the amputees, accomplishing tactile sensory feedback of prosthetics will improve their acceptance for the artificial limb (Antfolk et al., 2013).

Normally, our ability to feel the richness of our tactile environment relies on the cutaneous mechanoreceptors. These receptors detect and convert mechanical stimuli to corresponding electrical signals in the cutaneous nerve fibers. These signals can be delivered to the central nervous system, where they are further processed and interpreted as our tactile sensation (Gardner, 2010; Woo et al., 2015). Usually, thin-myelinated Aδ- or unmyelinated C- fibers mediate thermal and noxious stimuli which can produce pain sensation. The myelinated Aβ sensory afferents mostly innervate cutaneous mechanoreceptors, and are thought to produce an innocuous tactile sensation (Matsumoto et al., 2008). On the other hand, it was reported that some of Aβ fibers could innervate with the nociceptors (Djouhri and Lawson, 2004), and Aβ-fiber perception could be transmitted to spinal neurons, which originally received only Aδ- and C-fiber-mediated pain transmission, in animals with nerve injury (Matsumoto et al., 2008). Besides, Aβ fibers can activate a significant proportion of superficial dorsal horn GABAergic neurons for maintaining separation of touch and pain modalities (Daniele and MacDermott, 2009), and the electrical stimulation of the low-threshold Aβ afferent fibers may treat chronic neuropathic pain (Sdrulla et al., 2015). Then the Aβ fibers play an important role in tactile and pain sensation.

There are various kinds of ways to realize artificial tactile feedback. The mechanical tactile feedback can be obtained by using a skin pusher to convey tactile information. The typical example is the pneumatic system which was composed of pressure pads and a tube exerting pressure to the residual limb from the prosthetic fingers (Childress, 1980). The tactile feedback can also be achieved by electrical stimulation. By implanting microelectrode arrays in the somatosensory cortex, the electrodes can record the action potentials related to tactile sensation (Schwartz, 2004). Direct stimulation of the peripheral nerves is another way to elicit tactile feedback (Dhillon and Horch, 2005; Raspopovic et al., 2014). With a flat interface nerve electrode, the common nerve trunk can be selectively recruited (Tyler and Durand, 2002). Recently, the transcutaneous electrical nerve stimulation (TENS) was applied to accomplish tactile sensation for the amputee (Chai et al., 2015) and healthy subjects (Forst et al., 2015). Due to the non-invasiveness, the TENS could be promising in wide clinical applications to elicit tactile feedback. By attaching a surface electrode to the target skin area, various sensory afferents including Aβ, Aδ, or C fibers can be directly recruited without the activation of mechanoreceptors to produce artificial tactile or nociceptive sensation (Frahm et al., 2013). The excitatory state of sensory afferents such as Aβ nerve fibers can be modulated by different kinds of stimulating waveform parameters (Mahmud and Vassanelli, 2016).

To describe how the TENS evokes tactile neural activities and leads to psychophysical responses, the computational modeling is a feasible and efficient way, and a solid tactile nerve fiber model is the key to investigate the fiber excitation patterns. Although part of the Aβ nerve fibers contribute to pain sensation, they mostly play a significant role in feeling the pressure/light touch (Gardner, 2010). Most of the previous computational work about TENS focused on nociceptive Aδ- or C-type fibers (Yang et al., 2015), or motor nerve fibers (Kuhn et al., 2010; Goffredo et al., 2014). Although some papers established TENS model considering tactile sensory nerve fiber (Kajimoto et al., 2004; Mørch et al., 2011), the fiber model is passive without explicit ion channel distribution. The active tactile sensory nerve fiber model is more comparable to neurophysiological properties and important for TENS modeling. McNeal et al. established the first active multi-compartment cable model in 1976 to study the response patterns of action potentials. The model assumed the myelin sheath as a perfect insulator, and contained a few voltage-gated sodium and potassium channels at the nodes of Ranvier (McNeal, 1976). In 1995, Schwartz et al. recorded the action potentials and membrane currents in single human myelinated nerve fibers by means of the current and voltage-clamp techniques. They applied the obtained experimental data to the mathematical model derived from the Frankenhaeuser-Huxley equations (Schwarz et al., 1995). In 1999, Wesselink et al. implemented a human myelinated sensory nerve fiber model for direct spinal cord electrical stimulation (Wesselink et al., 1999). McIntyre et al. (2002) built a McIntyre-Richard-Grill (MRG) neuron model for mammalian motor nerve fibers on the foundation of Hodgkin-Huxley (H-H) model. In recent years, some researches adopted the MRG model as the sensory nerve fiber model in their computational modeling work (Bourbeau et al., 2011; Åström et al., 2015). However, the MRG model was derived from mammalian motor nerve fibers, and motor and sensory fibers were different in recovery cycle, action potential shape, and the properties of excitability (Howells et al., 2012). Consequently, a solid active tactile Aβ sensory nerve fiber is necessary for TENS modeling.

In our present study, we developed a novel three-dimensional (3D) computational model of TENS on the forearm to investigate the human subcutaneous Aβ tactile nerve fiber (TNF) excitation. The 3D model establishment was achieved by combining finite element model of the whole forearm, and the active TNF counterpart. The solid active tactile sensory nerve fiber model was optimized with integral ionic and morphological parameters based on the H-H model. In order to clearly figure out the excitability of TNFs under the TENS, the skin model was delaminated into multiple layers in terms of physiological structures. In addition, the effects of electrode size on the tactile sensation threshold were further compared between computational and psychophysical experimental results on healthy subjects. This novel 3D TENS model would lay a solid basis for optimization of surface electrodes in the transcutaneous electrical nerve stimulation.

## Methods

### The 3D finite element model

A 3D finite element model (FEM) of the forearm was created using COMSOL software (COMSOL Multiphysics 4.2a, Sweden). The FEM model was established to calculate the electrical potential distribution in the subcutaneous area during electrical stimulation. The stimulating waveform was biphasic charge-balanced cathodic-first current pulse. The FEM models were computed in the AC/DC module with a stationary electric current. According to the charge relaxation theory, the charge relaxation time τ is described as follows:

(1) $$\tau =\frac{\varepsilon }{\sigma }$$

where ε and σ are respectively the permittivity and conductivity of the tissues. In neural tissue, the values of these parameters are ε = 10−7 F/m and σ = 0.1 S/m (Gabriel et al., 1996a). As a result, the τ = 10−6 s, which is much smaller than the external time scale such as the stimulation pulse duration on the order of hundreds of microseconds. When applying current-regulated TENS, the influence of capacitance in the intermediate tissues can be neglected (Kuhn et al., 2009a).

**Figure 1:**
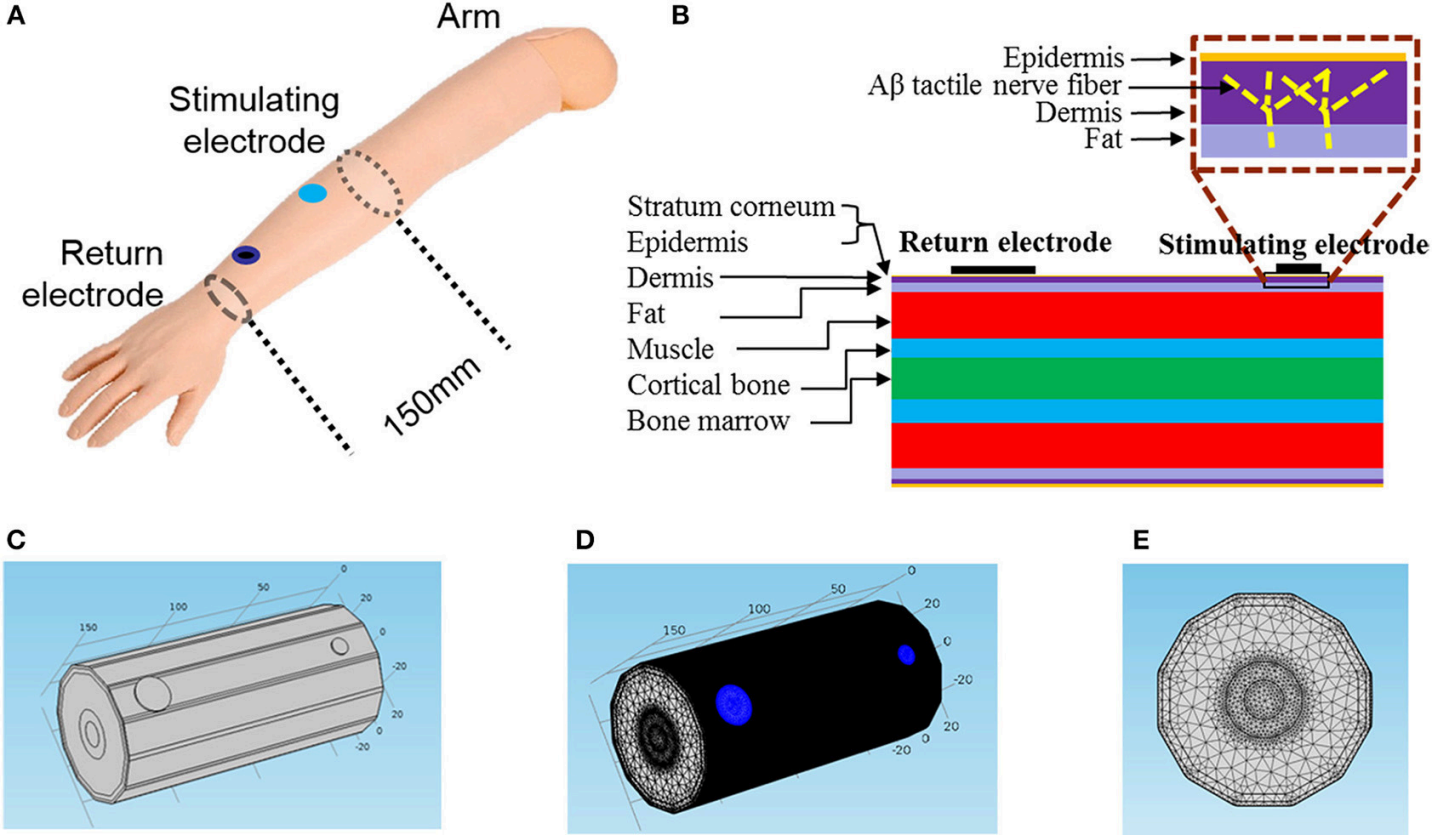
**Geometric structure of the 3D FEM for TENS. (A)** The schematic diagram of TENS on a subject's forearm. The distance between stimulating electrode and return electrode is 120 mm, and the return electrode diameter fixed to be 18 mm in diameter. The axial length of our total 3D FEM is 150 mm. **(B)** The layered structure of the forearm. In the box the bright dashed line illustrated the location of an Aβ tactile nerve fiber branching in the dermis layer. **(C)** Trimetric view of the 3D model. **(D,E)** Trimetric and front views of the model in extreme tiny tetrahedron mesh. The blue circle area denotes the two electrodes.

Mimicking the circumstance of psychophysical experiments on the forearm, a 150 mm-long cylindrical geometric structure with stimulating and return electrodes was modeled as shown in Figure 1. The computational model consisted of seven concentric layers: stratum corneum, epidermis, dermis, fat, muscle, cortical bone, and bone marrow. The detailed electrical properties and thicknesses were listed in Table 1.

**Table 1:** **Morphological and electric parameters in the FEM**.

	**Conductivity(S/m)**			**Thickness (mm)**
	**Min**	**Typical**	**Max**	
Stratum corneum	e-5^[a^^,^ ^b]^	2e-5^[a^^,^ ^b]^	e-4^[a^^,^ ^b]^	0.0290^[e^^,^ ^f]^
Epidermis(axial)		0.9500^[b^^,^ ^c]^		0.0600^[e^^,^ ^f]^
Epidermis(radial)		0.1500^[b^^,^ ^c]^		
Dermis(axial)	2^*^	2.5700^[b^^,^ ^c]^	3.8^*^	1.4110^[b^^,^ ^c]^
Dermis(radial)	1^*^	1.6200^[b^^,^ ^c]^	2.8^*^	
Fat	0.0017^[d]^	0.01^[d]^	0.1000^[d]^	2.5000^[d]^
Muscle(axial)	0.2000^[d]^	0.3333^[d]^	0.5000^[d]^	13.5000^[d]^
Muscle(radial)	0.0667^[d]^	0.1111^[d]^	0.1667^[d]^	
Cortical bone	0.0167^[d]^	0.0200^[d]^	0.0250^[d]^	6.0000^[d]^
Bone marrow	0.0667^[d]^	0.08^[d]^	0.1000^[d]^	6.5000^[d]^
Gel	0.0001^[d]^	0.0200^[d]^	1.0000^[d]^	1.0000^[d]^
Electrode(Pt)		8.9e6		0.0100

[a] Yamamoto and Yamamoto (1976);

[b] Gabriel et al. (1996b);

[c] Tavernier et al. (1993);

[d] Kuhn et al. (2009b);

[e] Neerken et al. (2004);

[f] Sandby-Moller et al. (2003) and the

* *notation denotes the values are adjusted for the sensitivity analysis. Through the thickness of the skin varied with many conditions, but the generally the total thickness is thought to be 1.5 mm (Kuhn et al., 2009a). Consequently the thickness of the dermis is determined by the stratum corneum and epidermis thicknesses and the whole skin thickness*.

In this model, the epidermis, dermis, and muscle layers are anisotropic, while the other tissues are regarded as isotropic. The electric scalar potential (V_FE_) in the FEM was described by Laplace's equation.

(2) $$-\nabla \cdot (\sigma \nabla V_{FE})=0$$

The σ is conductivity. By free tetrahedral meshing, there are more than 10 million elements in total with extremely tiny meshes due to the thin layers of epidermis and dermis. The computation was carried out using a computer workstation with an Intel Xeon CPU E5 and 64 GB of RAM.

### Subcutaneous Aβ tactile nerve fiber model

#### Morphological parameters

In our model, we developed Aβ nerve fibers with distribution density of approximate 2/mm^2^ under the skin (Lacour et al., 1991; Lesniak et al., 2014). As shown in Figure 1B, Aβ nerve fibers protruded from the muscle layer, and then aroused vertically to the skin which terminated in the dermis. This fiber length was 7 mm long, and was perpendicular to the skin except for the distal branching endings.

**Figure 2:**
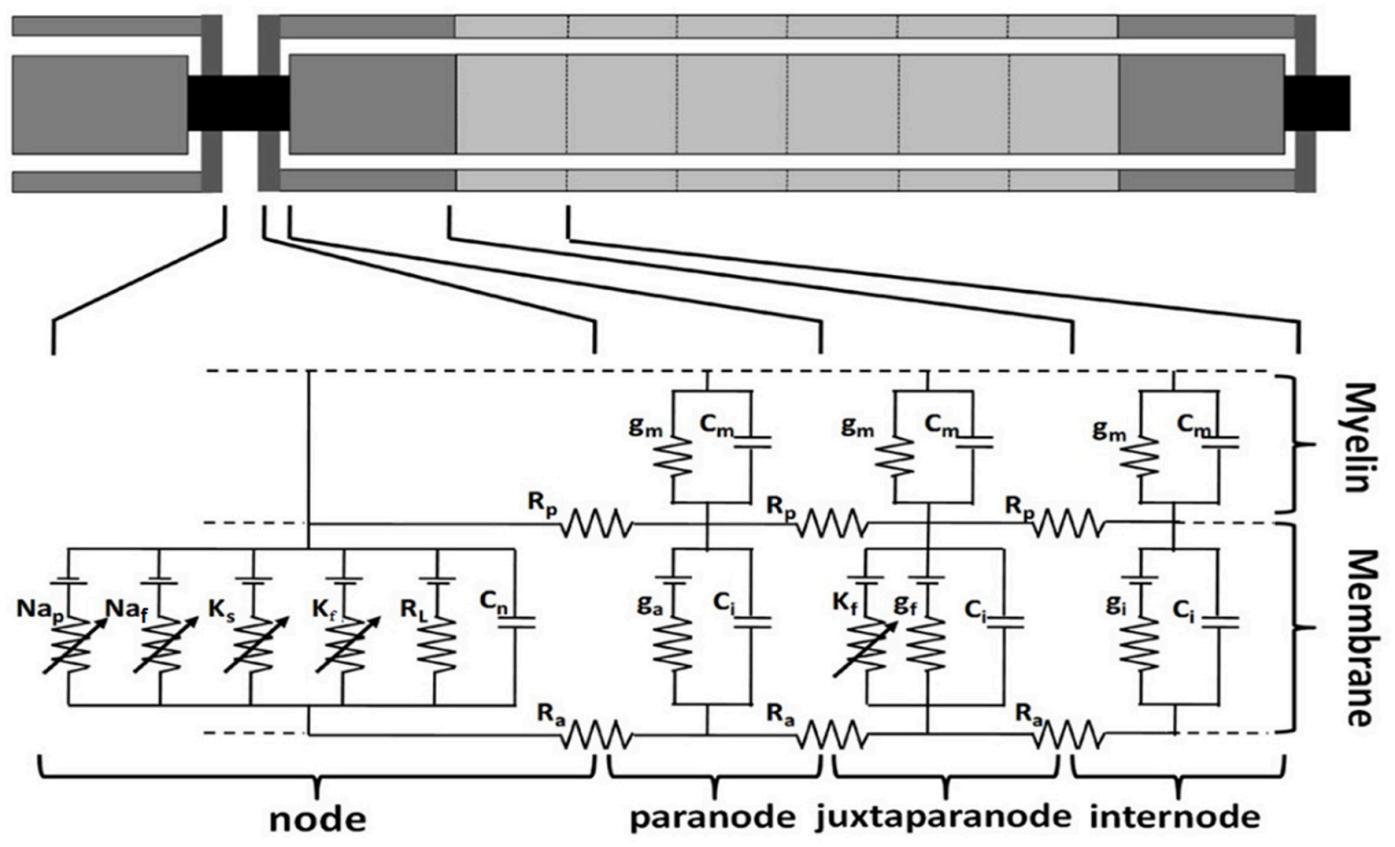
**Multi-compartment double cable model of a human sensory fiber**. The nodal membrane dynamics included fast sodium (Na_f_), persistent sodium (Na_p_), slow potassium (K_S_), fast potassium (K_f_), and linear leakage conductance (R_L_) in parallel with the nodal capacitance (C_n_). The juxtaparanode segments have fast potassium (K_f_) ion channel and linear leakage conductance (g_f_) in parallel with the internodal capacitance (C_i_). g_m_ and C_m_ are the conductance and capacitance separately in myelin sheath. R_p_ is periaxonal resistivity, R_a_ is axoplasmic resistivity. g_a_ and g_i_ are the conductance in paranode and internode segments separately (modified from Li et al., 2013).

The TNFs were developed including parallel double cable models with nodes of Ranvier, paranodal, juxtaparanodal, and internodal sections, and the myelin sheath, etc. based on previous studies (McIntyre et al., 2002; Li et al., 2013). These fiber models have similar structures with the same kinds of ionic channels but with different spatial distribution of ion channels as shown in Figure 2.

The internodal section consists of two paranodal myelin attachment segments, juxtaparanodal segments and six internodal segments such that relationship remains valid for the compartments in all models. The Node-to-node length represents the distance between two adjacent Ranvier node centers, as shown in Equation (3).

(3) $$\begin{array}{l} \text{Node}-\text{to}-\text{node}\;\text{ length}=\text{nodal}\;\text{ length}\\ \;+\;2\times \text{paranodal}\;\text{ length}\;\\ \;+\;2\times \text{juxtaparanodal}\;\text{ length}\;\\ \;+\;\;6\times \text{internodal}\;\text{ length}\; \end{array}$$

The models were built in NEURON v7.3 environment, and solved using backward Euler implicit integration with a time step of 0.001 ms.

**Figure 3:**
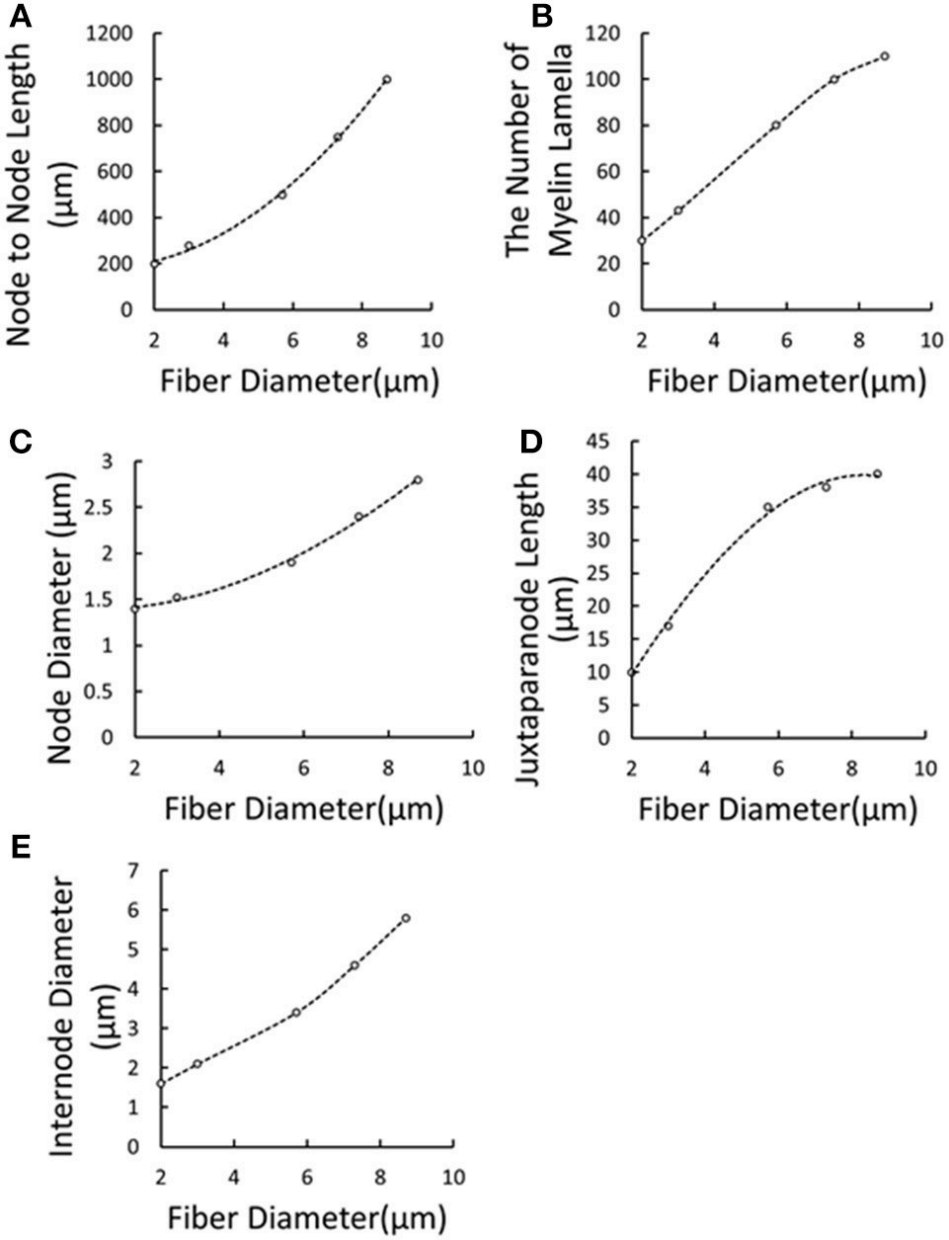
**Extrapolation and interpolation of morphological parameters using cubic spline fitting**. **(A)** Node to Node Length vs. Fiber Diameter; **(B)** The Number of Myelin Lamella vs. Fiber Diameter; **(C)** Node Diameter vs. Fiber Diameter; **(D)** Juxtaparanode Length vs. Fiber Diameter; **(E)** Internode Diameter vs. Fiber Diameter. Dots denoted reported experimental data (McIntyre et al., 2002, 2004; Sotiropoulos and Steinmetz, 2007) and dash lines represented the regression curves.

It was reported that the axonal diameter of Aβ fibers was about 10 μm (McGlone and Reilly, 2010), while such big diameter was always measured on the ulnar or median nerve trunk. It was found that some mechanoreceptor-innervated nerve fibers such as Ruffini corpuscle afferent axons measured 2–4 μm in diameter (Halata et al., 1985). And, the 3 μm was most common diameter in the superficial cutaneous layer (Provitera et al., 2007). So the diameter of cutaneous Aβ nerve fiber is much compatible to the sural nerve fiber with 3.11 μm (Van Veen et al., 1995). The number of myelin lamellae and axon diameter was not constant, but decreased toward the distal end. Caruso G et al. found that fiber diameters in the skin were at least 3 times thinner than the respective proximal segments in the nerve trunk (Caruso et al., 1992). Besides, Provitera denoted that the subcutaneous fiber diameter should be about 2–8 μm (Provitera et al., 2007). As a result, under TENS of the forearm, the tactile Aβ afferents were selected as small myelinated nerve fibers in our present work. However, no detailed morphological parameters were reported adaptable to small Aβ fibers. The morphological parameters for the fibers were listed in Table 2. Some parameters varied with diameters, and the others were fixed according to the experimental data. So we used numeric interpolation and extrapolation to estimate the morphological parameters of Aβ tactile fibers with different diameters as shown in Figure 3. Then, the varied morphological properties can be determined including node-to-node length (X1), the number of myelin lamella (X2), node diameter (X3), juxtaparanode diameter (X4), internode length (X5).

**Table 2:** **Geometric parameters for Aβ TNF**.

**Variables**	**Value**
Node-to-node length^a^	X_1_μm
The number of Myelin lamella^a^	X_2_
Node length	2 μm
Node diameter^a^	X_3_μm
Paranode length	3 μm
Paranode diameter^a^	X_3_μm
Paranode psw^b^	0.002 μm
Juxtaparanode length^a^	X_4_μm
Juxtaparanode diameter^a^	X_5_μm
Juxtaparanode psw^b^	0.004 μm
Internode length^c^	(X_1_-2X_4_-7)/6 μm
Internode diameter^a^	X_5_μm
Internode periaxonal space width	0.004 μm

*These fixed values are taken from previous studies. (McIntyre et al., 2002, 2004; Sotiropoulos and Steinmetz, 2007)*.

a *Values of these parameters are all dependent variables of fiber diameters (Figure 3)*.

b *psw: periaxonal space width*.

c *Derived from Equation (3)*.

#### The ion channel and membrane dynamics in the Aβ TNF fiber model

To simulate the behavior of the axon under electrical stimulation, the model we implemented contains both linear and nonlinear membrane dynamics. There are many kinds of voltage-gated ion channels in the axon, and the ion channels located in the axonal membrane carry out action potential initiation and conduction by governing the amplitude and shape of the unitary spike and pattern of repetitive firing (Debanne et al., 2011). We developed a kind of human peripheral TNF model based on the double cable model structure (McIntyre et al., 2002; Li et al., 2013). The node Ranviers consisted of slow K^+^, fast K^+^, fast Na^+^, and persistent Na^+^ conductance. The juxtaparanodal consisted of fast K^+^ conductance in parallel with the membrane and leakage conductance. The dynamics of the nodal and internodal ion channels (Equations in Appendix) were based on the experimental data of sensory nerve fibers (Howells et al., 2012). The maximum density of fast K^+^ in the sensory nerve fiber (Howells et al., 2012) is far more than that in the motor fiber (McIntyre et al., 2002), so it was incorporated to our TNF model. Then the novel Aβ tactile sensory nerve fiber model was achieved. All of the parameter values were derived from the reported experimental data, and dynamic equations form for sodium ionic channel was based on ionic conductance. The electrical parameters of the Aβ TNF model are shown in Table 3.

**Table 3:** **Electrical parameters for the cable model**.

**Model electrical parameters**	**Accepted values**
Nodal capacitance (c^n^)	2.8 μF/cm^2^^[a]^
Internodal capacitance (c^i^)	2.8 μF/cm^2^^[a]^
Myelin capacitance (c^m^)	0.1 μF/cm^2^^[b]^
Axoplasmic resistivity (R^a^)	33 Ωcm^[a]^
Periaxonal resistivity (R^p^)	33Ωcm^[a]^
Myelin conductance (g^m^)	0.001 S/cm^2^^[b]^
Myelin attachment conductance (g^m^)	0.001 S/cm^2^^[b]^
Paranodal conductance (g^a^)	0.0001 S/cm^2^^[b]^
Internodal conductance (g^i^)	0.0001 S/cm^2^^[b]^
Max transient Na^+^ conductance (Na^f^)	3^[b]^
Persistent Na^+^ conductance (Na^p^)	0.0321^[d]^
Max slow K^+^ conductance (K^s^)	0.06 S/cm^2^^[a]^
Max fast K^+^ conductance (K^f^)	0.03 S/cm^2^^[a]^
Max juxtaparanodal fast K^+^ conductance (K^f^)	0.3 S/cm^2^^[d]^
Nodal leakage conductance (R^L^)	0.06 S/cm^2^^[a]^
Na^+^ Nernst potential (E^na^)	43.7 mV^[a]^
K^+^ Nernst potential (E^k^)	−84 mV^[c]^
Leakage reversal potential (E^L^)	−84 mV^[c]^
Rest potential (V^rest^)	−80 mV^[d]^

[a] Wesselink et al. (1999);

[b] McIntyre et al. (2002);

[c] Schwarz et al. (1995);

[d] *Howells et al. (2012)*.

### The combined 3D TENS modeling

This combined 3D TENS model is accomplished by integration of the forearm FEM model implemented in COMSOL and TNF model developed in NEURON. Stimulating currents were applied to the surface electrode on the forearm FEM model in COMSOL, and then the subcutaneous spatially interpolated electric potentials V_FE_ in COMSOL were exported to NEURON as the corresponding extracellular voltages Ve (Ve = V_FE_) of the TNFs. Thus, the excitability of the TNFs under TENS can be quantitatively determined, and the excitation threshold was defined as the minimum current intensity to recruit one fiber.

### Model validation

By adopting typical FEM model parameters in Table 1 and TNF properties in Tables 2, 3, the 3D TENS model was validated in terms of conduction velocity of the TNFs and the strength-duration relationship of the surface electrical stimulation. The conduction velocities were computed in NEURON corresponding to different fiber diameters ranging from 2 to 8 μm. The curves of conduction velocity vs. fiber diameters were compared with previously reported sensory fiber modeling results (Wesselink et al., 1999) and experimental data (Van Veen et al., 1995). In addition, with the stimulating frequency of 50 Hz and pulse duration of 200 μs, the strength-duration relationship in our 3D TENS model were achieved for a given Φ-9 mm electrode and an Φ-3 μm Aβ nerve fiber with typical electrical properties. The stimulating pulse durations were selected increasing from 10 μs to 1 ms, and the corresponding threshold current intensities were obtained. Strength-duration curves were further compared among our computational, psychophysical experimental and reported modeling results.

### Forearm model parameter sensitivity analyses

The tactile sensation threshold varied between different individuals under TENS. To potentially explore what caused the threshold variance between individuals, we made several sensitivity analyses on the tissue properties. The thickness and conductivity in fat, muscle and dermis layers were changed to explore what tissue properties would contribute most to the TENS difference. As shown in Table 1, the fat conductivity changed between 0.0017 and 0.1 S/m, the muscle conductivity (axial) between 0.2 and 0.5 S/m. The muscle conductivity (radial) between 0.0667 and 0.1667 S/m, the dermis conductivity (axial) between 2 and 3.8 S/m, and the dermis conductivity (radial) between 1 and 2.8 S/m. The corresponding thickness variation ranges were as follows: 1.5–4 mm for Fat layer, 1.2–1.7 mm for Dermis layer, and 8–18 mm for Muscle layer. Many combinations can be obtained with the ranges from different parameters. In order to get the threshold current relationship with different conductivities or thicknesses, the specific parameter changes within a variation range, and the remaining geometric and electrical properties were kept constant as the typical values in Table 1.

### Threshold currents under the 3D TENS modeling

Based on this validated 3D TENS model, the effects of electrode size and fiber diameters on the threshold current intensity were investigated. Fiber diameters were selected from 2 to 8 μm with a step of 1 μm, the surface electrode size from 5 to 12 mm, pulse duration from 10 μs to 1 ms. With the Φ-9 mm surface electrode and Φ-3 μm fiber, the spatial distribution of threshold current intensity below the surface electrode was obtained, and the fiber excitation characteristics were further observed under different stimulating currents.

### Psychophysical experiments

The psychophysical experiments were carried out for five consecutive days on six healthy subjects (aged 22–30 years, 3 males, 3 females) without clinical or neuro-physiological disorders. All subjects were provided the informed written consent prior to commencement of the experiment. All experiments were conducted in accordance with the Declaration of Helsinki and approved by the Ethics Committee of Human and Animal Experiments of School of Biomedical Engineering at Shanghai Jiao Tong University (No. 2016012). In our experiments, we used Master-9 Pulse Stimulator with two isolators (Iso-Flex, A.M.P.I. Company, Israel) to generate biphasic charge-balanced cathodic-first stimulating current pulses. The gold stimulating electrode was placed on the volar part of forearm 2 cm from the proximal wrist crease and the return electrode approximately 12 cm apart from the stimulating electrode on the same side. The same-size thin-layer conductive gel was adhered to the stimulating electrode during the TENS. An Φ-25 mm non-woven surface electrode (Shanghai Kongren Medical, Inc.) was used as the return electrode. The subjects sit in a chair with a comfortable position, and the small current intensities were adopted to electrically stimulate the forearm skin through the electrode to produce the pressure sensation. The experiments started only when the room temperature was stable at 27°C. During all the experiments, the subjects were given 5-min relaxation every 20 min to assure the comfort during the whole psychophysical experiments.

The tactile perception thresholds were defined as the least current pulse intensity or amplitude that a subject could detect, and was determined by using a staircase paradigm. The pulse amplitude was first increased with a step of 0.1 mA, and then a step of 0.01 mA to estimate the potential perception threshold current. Immediately after each stimulation, the subjects were required whether or not the stimulus was perceived. Tens of stimulating trials were conducted near the potential threshold currents, and the exact perception threshold was determined in terms of 50 percent level of the trails. In the psychophysical experiments, the effects of diameters of the gold stimulating electrode (5–12 mm) and the pulse duration (50–400 μs) on the tactile perception threshold were also investigated.

The effects of individual subject difference on the perception threshold were also statistically evaluated in the psychophysical experiments. The independent variable was “individual subject,” and the dependent variable was “perception threshold.” A one-way analysis of variance (ANOVA) was performed and the *F*-test was used to find out the significance of the independent variable. The significance level was chosen as 0.05.

## Results

### Model validation

**Figure 4:**
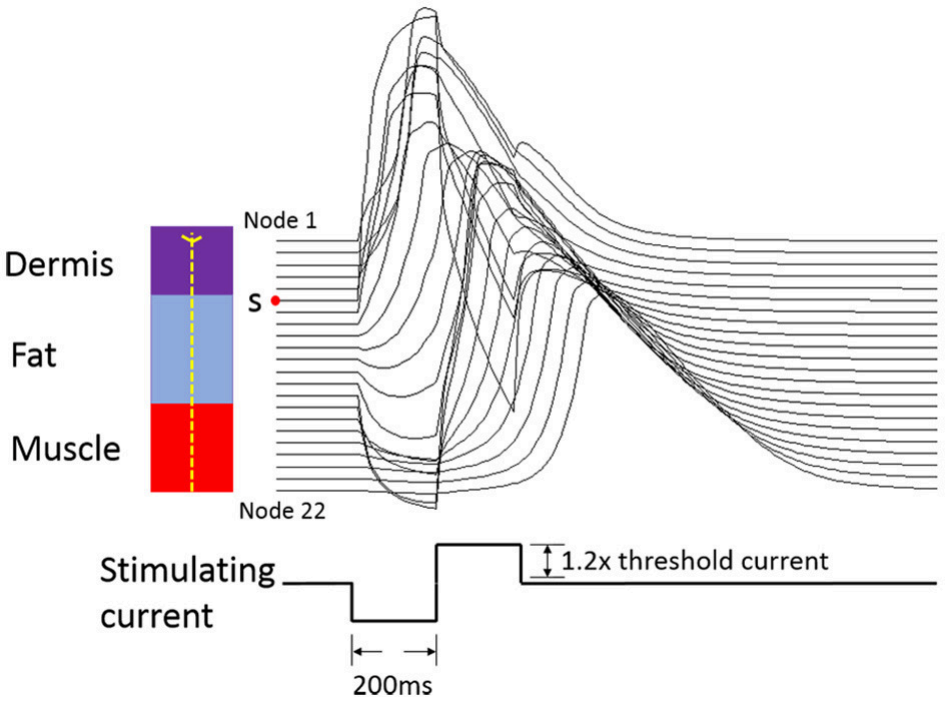
**Conditions for action potential propagating under biphasic charge-balanced cathodic-first current pulse stimulation**. The 1.2× means the stimulation current amplitude was 1.2 times the threshold current. The red point S is the initial node for the action potential. The left panel shows the corresponding position for each Ranvier node in an Aβ tactile fiber.

**Figure 5:**
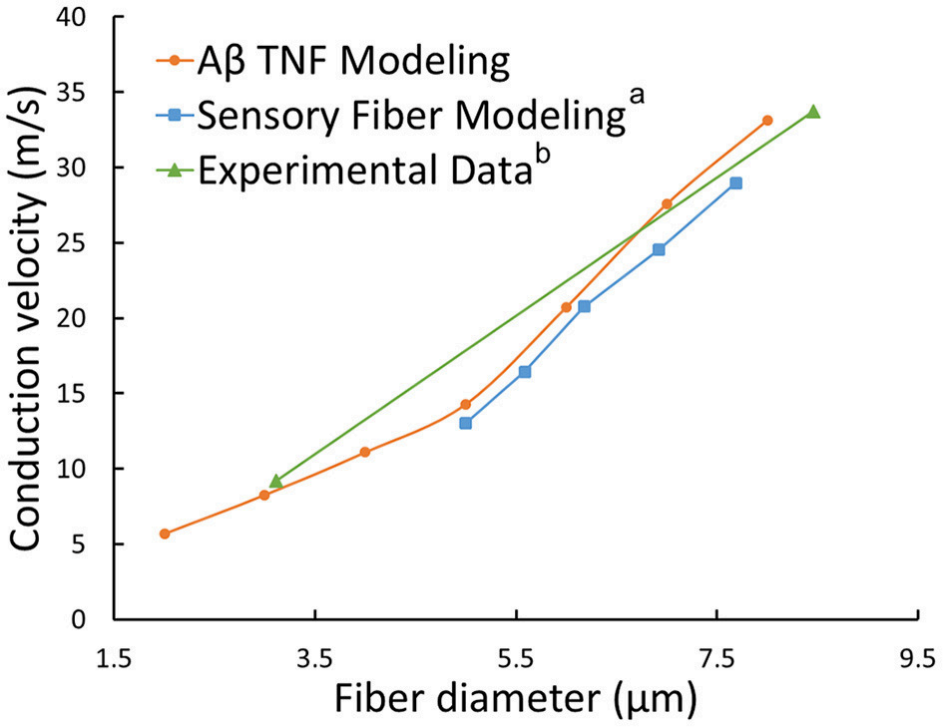
**Relationships between conduction velocities and fiber diameters: our TNF modeling (orange line), sensory fiber modeling (a Wesselink et al., 1999) and experimental data (b Van Veen et al., 1995)**.

The typical propagating characteristics of action potentials in an Φ-3 μm Aβ nerve fiber were shown in Figure 4 under biphasic charge-balanced cathodic-first current pulse stimulation. The pulse duration was 200 μs, and the stimulating intensity was 2.84 mA which is 1.2 times the stimulating threshold. The action potential was excited at node S, and then propagated along both directions of the fiber. The other nodes were first hyperpolarized, and then depolarized when the propagating action potential arrived. The conduction velocity of this typical TNF was 8.27 m/s in terms of Figure 4. The conduction velocities corresponding to varied fiber diameter were also shown in Figure 5. With the fiber diameter increasing from 2 to 8 μm, the corresponding conduction velocity increased from 5.68 to 33.14 m/s, which matched well with experimental data (Wesselink et al., 1999), and previously published modeling results in terms of small-diameter tactile sensory nerve fibers (Van Veen et al., 1995). The conduction velocity is dependent on a host of factors such as myelin sheath thickness and internodal length (Waxman, 1980). These morphological parameters were interpolated from various existing models as shown in Figure 3. Other parameters such as axoplasmic resistivity which have various alternatives were also optimized to match the experimental results. The ultimate optimal parameters as for the morphological parameters and electrical properties were denoted in Tables 2, 3, respectively.

**Figure 6:**
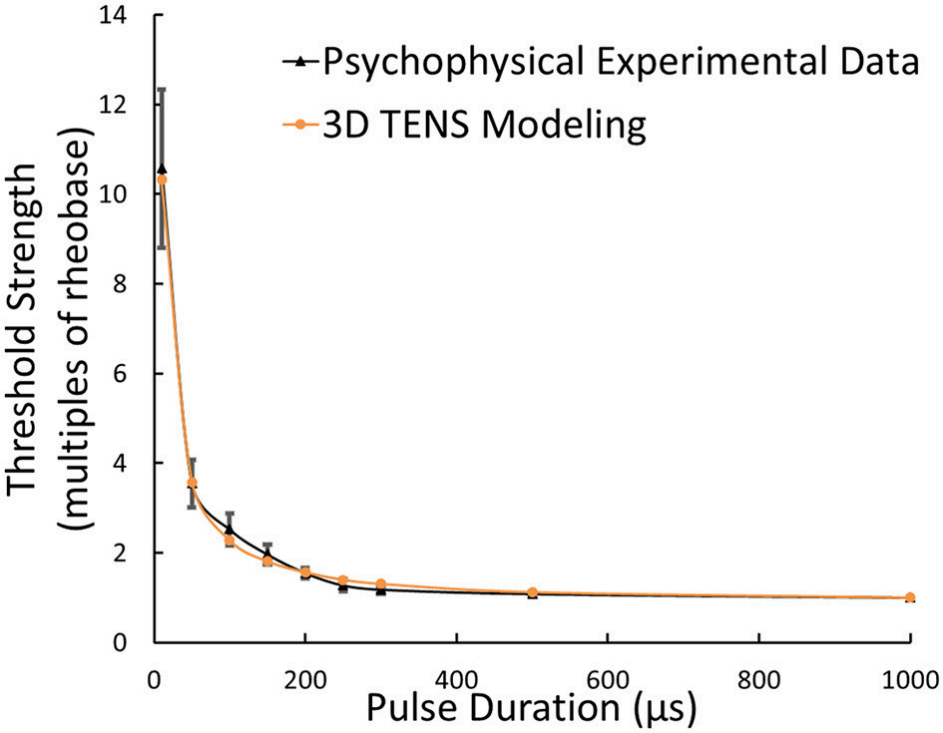
**Strength-duration relationship from 3D TENS Modeling, psychophysical experimental data; Error bars showed the standard deviations of experimental results**.

With standard 3D TENS model parameters selected, the strength-duration relationship was illustrated in Figure 6, where the Φ-3 μm Aβ nerve fibers and Φ-9 mm stimulating electrodes were adopted. As illustrated in Figure 6, multiples of the rheobase current was selected to describe the stimulus threshold. With pulse duration enhancing, the threshold decreased sharply within short pulse durations and maintained stable at durations larger than 500 μs. The threshold currents for tactile perception were obtained based on psychophysical experiments from 6 healthy volunteers. Figure 6 showed that the strength-duration tendency was closely consistent with our psychophysical experimental results.

### Forearm model parameter sensitivity analyses

**Figure 7:**
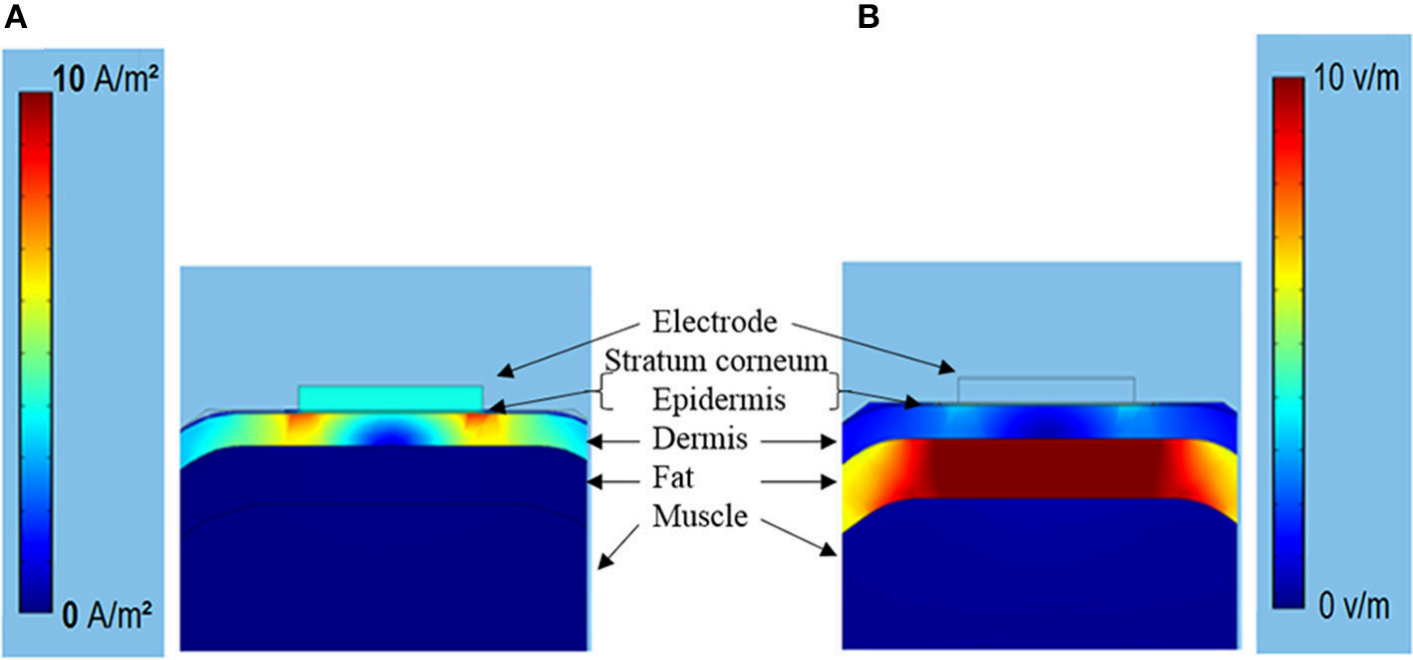
**(A)** The current density (0–10 A/m^2^) distribution under the TENS with stimulating current of 0.5 mA. **(B)** The corresponding electrical field strength distribution (0–10 V/m).

**Figure 8:**
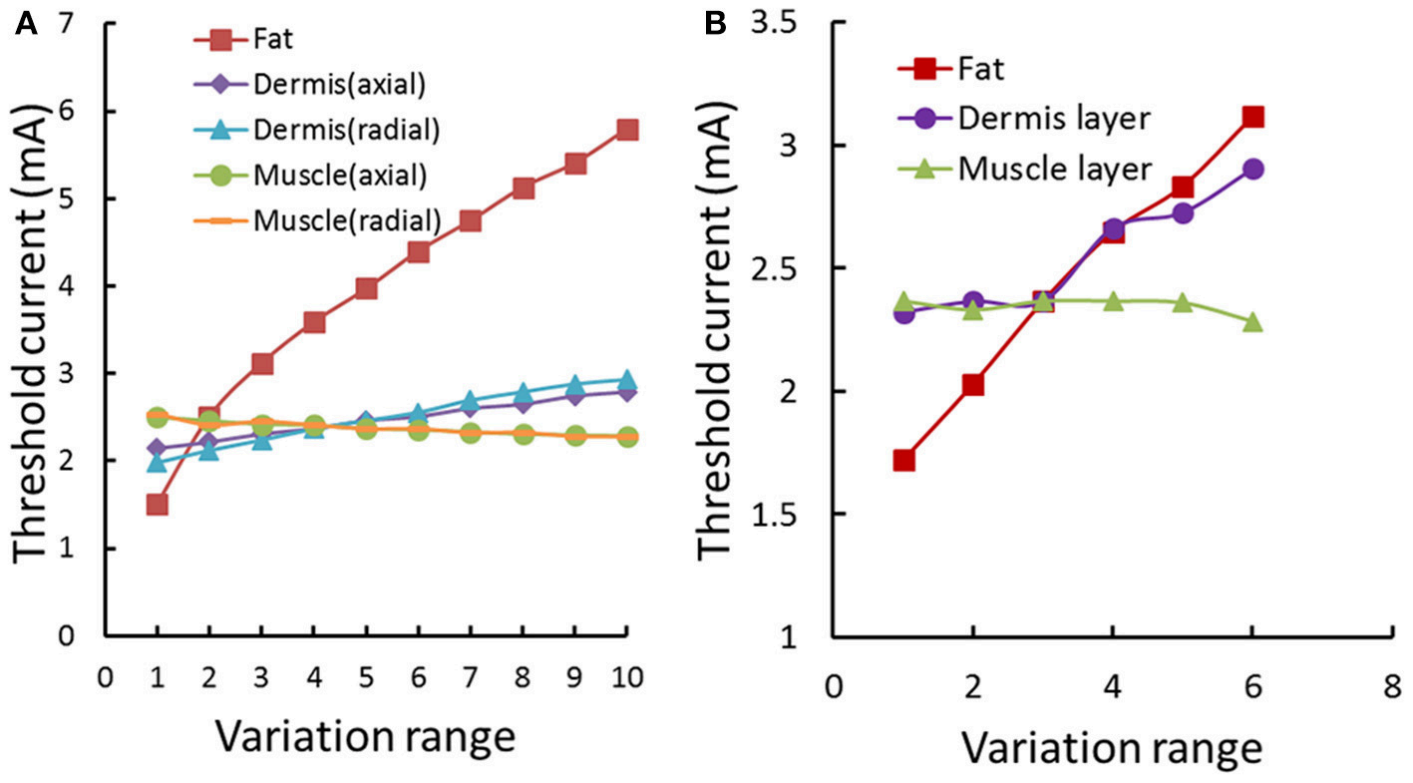
**Threshold current variation under the forearm model sensitivity analysis. (A)** The threshold current variation with electrical conductivity changing of human arm tissues. The different tissue conductivities varied in a given range as shown in Table 1. The Fat conductivity of 0.0017–0.1 S/m; The muscle conductivity (axial) of 0.2–0.5 S/m; The muscle conductivity (radial) of 0.0667–0.1667 S/m; The dermis conductivity (axial) of 2–3.8 S/m; The dermis conductivity (radial) of 1–2.8 S/m. The range of conductivity was segmented into 10 evenly spaced parts. **(B)** The threshold current variation with thickness of human arm tissues. The fat thickness is between 1.5 and 4 mm; The muscle thickness is between 8 and 18 mm; The dermis thickness is between 1.2 and 1.7 mm. The range of the thickness in each tissue was segmented into 6 evenly spaced parts.

Figure 7 showed the typical subcutaneous distribution of current density and electrical field strength during the TENS. The current density reached the maximum in the dermis layer, especially near the electrode edge. On the contrary, the strongest electrical field occurred in the fat layer due to the lower conductivity. The properties in dermis and fat layers may play an important role in the tactile sensation threshold. Figure 8A showed that the threshold current rose with the increasing electrical conductivity of fat and dermis layers while descending with the increasing electrical conductivity of the muscle layer. Within the given variation range shown in Table 1, the threshold current was most sensitive to the electrical conductivity of fat tissue. Figure 8B showed that the threshold current rose with the increasing thickness of fat and dermis layers, while descended a bit when increasing the muscle thickness during the given range. The thickness of fat and dermis layers showed obvious effect on the threshold current.

### Threshold currents under the TENS

**Figure 9:**
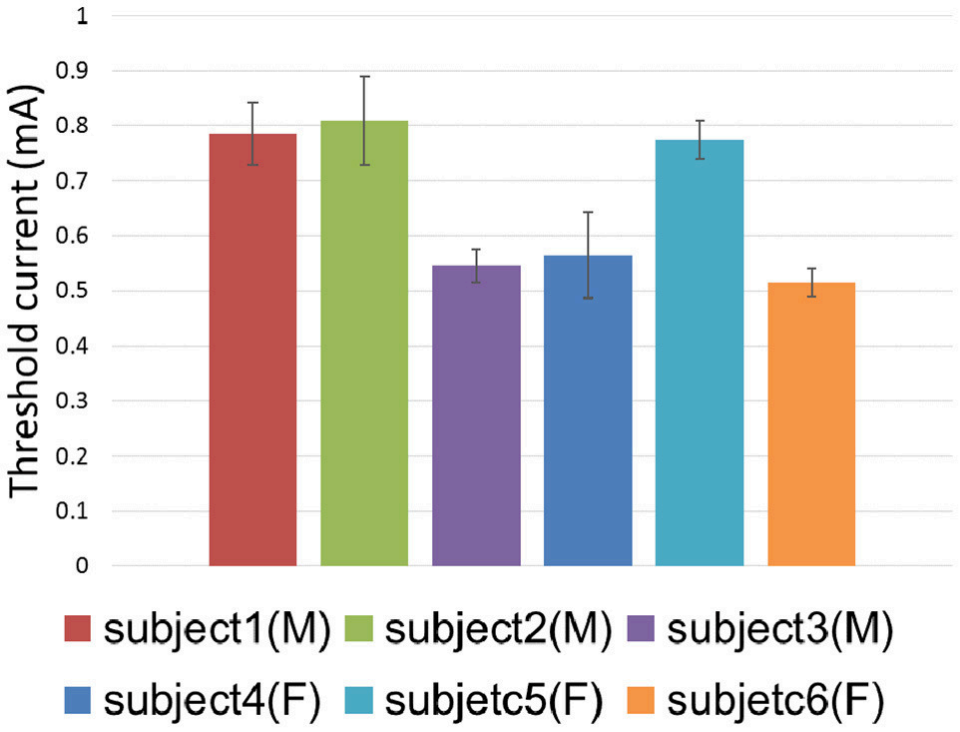
**Threshold current distribution among different subjects**. Error bars showed the standard deviations of psychophysical experimental results.

The threshold currents were further investigated by means of both the 3D TENS computation and psychophysical experimental work. The threshold currents in terms of 6 subjects were shown in Figure 9. The one-way ANOVA results indicated that the different subject showed a significant variance on the tactile perception threshold [*F*_(5, 24)_ = 24.565, *p* < 0.0001]. The tactile sensation threshold were between 0.5 and 1.5 mA in our psychophysical experiments, and the corresponding strength-duration relationship was illustrated in Figure 6, which showed similar tendency to that of our modeling results.

**Figure 10:**
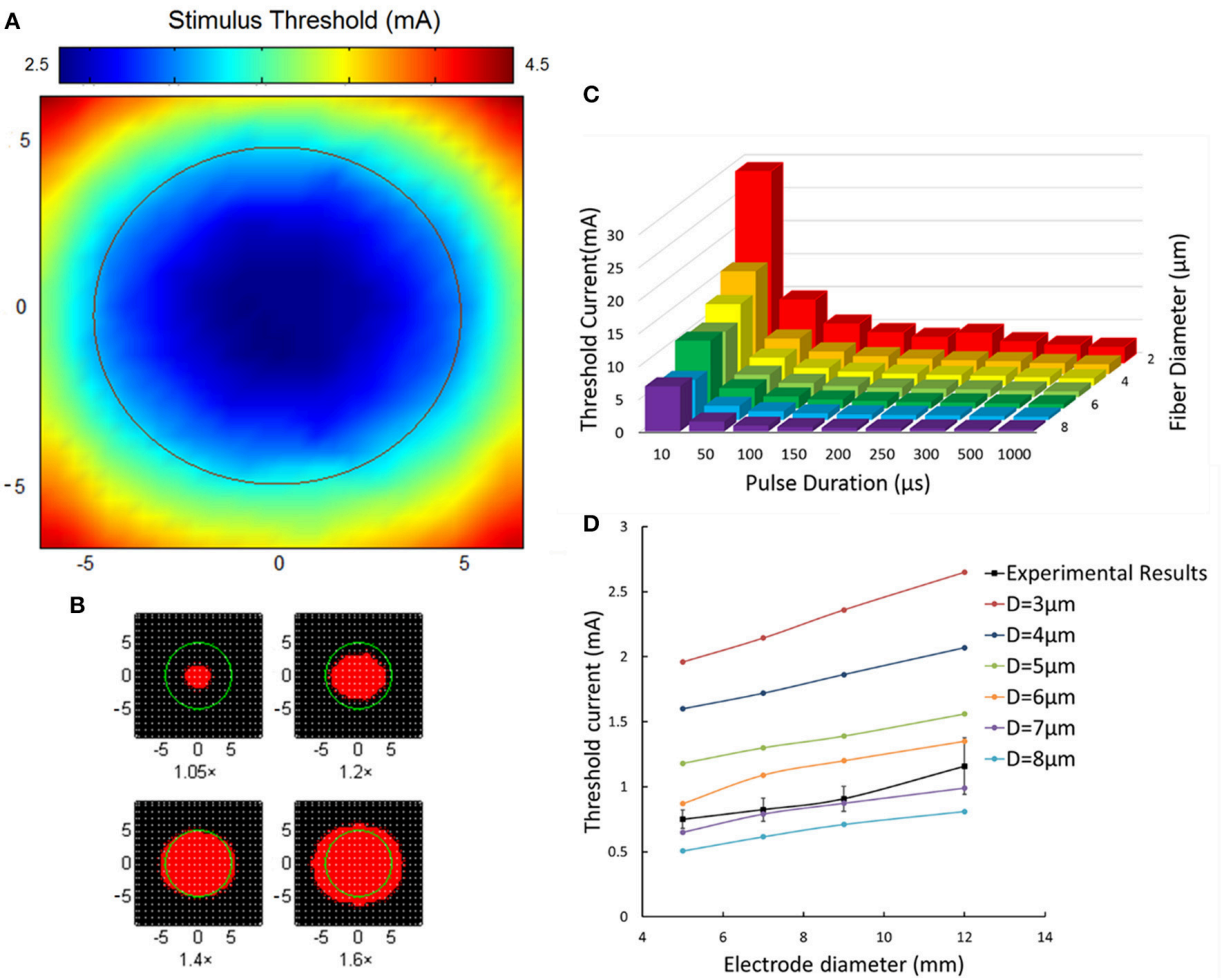
**Aβ tactile nerve fiber excitability under different electrical stimulation. (A)** The nerve excitation threshold distribution under the TENS (Φ-9 mm electrode). The color map corresponds to the threshold distribution. The middle circle corresponds to the 9 mm stimulating electrode. **(B)** The nerve excitation area (red) varies with different stimulating current. The 1.05× means the stimulating current was 1.05 times the least threshold current in the skin. The same as 1.2×, 1.4×, and 1.6×. **(C)** The fiber excitation thresholds under different pulse duration with different-diameter fibers. **(D)** The threshold current for 3–8 μm diameter axons under different size of electrode stimulation. The dashed line corresponds to the experimental threshold current density. Error bars show the standard deviations of experimental results.

Figure 10A showed the spatial distribution of threshold current in terms of Φ-3 μm axons under the Φ-9 mm stimulating electrode. It was clear that the least excitation threshold occurred axially below the stimulating electrode corresponding to one fiber excitation. The threshold increased apparently with a further distance from the electrode center. The threshold current below the edge of the electrode almost two times than that of the electrode center. Figure 10B showed that when stimulating current increased a little more than the threshold, the fiber excitation area extended greatly. When applying current intensity 1.4 times the threshold, the excitation area was close to size of the Φ-9 mm stimulating electrode.

Figure 10C illustrated the nerve excitation threshold during 3D TENS computation for different axon diameters and pulse durations. Larger nerve fibers were recruited at lower current amplitudes. The threshold current intensity decreased with fiber diameter from 2 to 8 μm, and varied sharply with small pulse duration stimulation. For instance, with pulse duration of 50 μs for Φ-3 μm fibers, the threshold was 1/3 compared with that at 10 μs duration. While, with tenfold variation of pulse duration from 100 to 1,000 μs, the threshold current at 1,000 μs only decreased to be about 1/2 at 100 μs. Besides, at short durations, the threshold difference among different-size fibers was more obvious than that of long durations. For example, the threshold currents at 10 μs for Φ-2 μm fibers were 22 mA bigger than that of the Φ-8 μm fibers, and the difference decreased to be 3.3 mA at 200 μs correspondingly.

In addition, under typical pulse duration of 200 μs and frequency of 50 Hz, the effects of different fiber diameters and stimulating electrode sizes on the threshold current were further compared between 3D TENS computational results and the psychological experimental data in Figure 10D. Computational results showed that the threshold current amplitude increased gradually with electrode size enlarging from 5 to 12 mm. With fiber diameter increasing from 3 to 8 μm, the threshold current decreased. The black square line depicted the psychophysical experimental results averaged from 6 healthy volunteers with similar changing tendency. This line fitted closely to the changing tendency of the Φ-7 μm fiber in the computational results.

## Discussion

In this study we developed a novel general 3D TENS computational model which could guide theoretical estimation of the recruitment of tactile afferent fibers. Although sensory nerve model had been studied for many years (Schwarz et al., 1995; Wesselink et al., 1999; Smit et al., 2009; Howells et al., 2012), the specific active model of tactile sensory fibers had not been studied in detail during the TENS. Our tactile nerve fiber model adopted the widely-accepted double-cable-layer model structure. The ionic types and kinetics parameters in this TNF model were derived from the recent study results (Howells et al., 2012), which were different from the previous parameters associated with the motor fiber model (McIntyre et al., 2002). The slow potassium channels in the internode were removed due to the low density distribution (Howells et al., 2012). The other electrical parameters were optimized by mathematical fitting based on the previous separate physiological experiments. This novel fiber model was validated by comparing the velocity vs. diameter and strength vs. duration relationships between our modeling and experimental results. As for the finite element modeling work, the 3D forearm was modeled, and the skin structure was finely layered into stratum, epidermis and dermis layers. This fine structure would be more practicable in comparison with the non-layered skin structure in some previous work (Kajimoto et al., 2004; Kuhn et al., 2009a). Based on the combined 3D TENS model consisting of finite element model of forearm and tactile nerve fiber model, the threshold current variation under TENS in terms of different electrode size and fiber diameters were investigated. The TENS computational results showed high consistency with psychophysical experimental results among 6 healthy volunteers.

The 3D TENS model were validated based on the action potential propagation, conduction velocity vs. fiber diameter relationship, and the strength-duration relationship. The action potential was excited in the Ranvier node S, and then spread along both directions of the axon. The node S is the closest Ranvier node to the dermis-fat junction where the electrical field intensity was enhanced due to the fat layers. The action potentials showed similar propagating characteristics under cathodic current stimulation with the reported computational result (Rattay, 1999). The conduction velocity matched well with reported experimental (Van Veen et al., 1995) and other sensory nerve fiber modeling work (Wesselink et al., 1999).

In strength-duration relationship, the chronaxie was not adopted in our model validation for the reason that many factors can affect the accuracy of the chronaxie measurement (Geddes, 2004), and then we used multiples of rheobase as our comparison standard between modeling and psychophysical experimental work in Figure 6. It is more meaningful to have the specific model parameters for each subject which provided similar trend between the threshold current vs. duration. While, Figure 6 does not show the relationship between the threshold current and duration, and the Y-axis represents the threshold strength with the unit of multiples of rheobase, which was equal to the threshold current divided by the respective rheobase current. From the sensitivity analyses results, it is clear that conductivity and thickness of the fat layer play a key role in the variation of threshold current during TENS. So the specific fat properties for each subject should be incorporated into the model parameters to have similar trends between the threshold current and duration among different subject. This work will be carried out in the future work.

Threshold current intensities showed significant difference between individuals in our psychophysical experiments, which was consistent with previous studies (Larkin et al., 1986; Ara et al., 2012). The sensitivity analyses of the electrical properties in the forearm tissue clearly showed that both the thickness and electric conductivity in the fat layer greatly affected the variation of threshold current intensities. It could be inferred that fat would play an important role in the tactile perception threshold in the psychophysical experiments.

As shown in Figure 10D, the relationship of threshold currents vs. electrode sizes in the psychophysical experiments was most comparable to the Φ-7 μm Aβ fibers in the computational modeling results. It was indicated that Aβ nerve fibers with diameters of no less than 7 μm would be more associated with the comfortable tactile perception for the subjects. Nevertheless, there still exists modeling limitation. Although the strength-duration trend matches well in Figure 6, the threshold strength only represented multiples of rheobase, and the threshold current for a 3 μm diameter fiber is far more than the experimental values in Figure 10D. As a result, in our forthcoming work, a coefficient might be included to the computation of the threshold current, and to achieve similar threshold current-duration trends between computational and experimental results.

By establishing this novel 3D TENS model, we would directly observe the natural tactile sensation activities under the electrical stimulation. Some other issues can also be solved based on this model such as the relationship of nerve fibers activation with the two point discrimination under electrical stimulation (Solomonow et al., 1977) and the influence of nerve fiber regeneration or degeneration on the electrical stimulation (Simpson et al., 2013; Hebert et al., 2014).

## Conclusion

In this paper, we introduced a novel Aβ TNF cable model used for TENS. The model is validated with previous studies and psychological experiments. At the same time, by combining 3D FEM and TNF modeling methods, we developed a novel 3D TENS model to explore the Aβ TNF excitatory threshold currents under TENS. This novel 3D TENS model was validated by comparing computational and experimental results in terms of conduction velocity vs. fiber diameter and strength-duration relationships. Then we investigated the effects of electrode size and fiber diameters on the threshold current intensities. The computational results illustrated that the larger the nerve fibers, the lower the threshold current intensities. These computational results corresponded to our psychophysical experimental data on six healthy subjects. This presented novel 3D TENS model would further guide the generation of artificial tactile sensation based on the surface electrodes.

## Ethics statement

This study was carried out in accordance with the recommendations of the Ethics Committee of Human and Animal Experiments of the Med-X Research Institute at Shanghai Jiao Tong University with written informed consent from all subjects. All subjects gave written informed consent in accordance with the Declaration of Helsinki. The protocol was approved by the Ethics Committee of Human and Animal Experiments of the School of Biomedical Engineering at Shanghai Jiao Tong University.

## Author contributions

XS contributed to the design of the overall experiment and simulation approaches; KZ conducted the simulation and psychophysical experiments. KZ wrote the first draft and XS also contributed to the whole manuscript revision. LL contributed to fiber model programming, and the manuscript revision. XW contributed to fiber model programming. All authors were active in the editing and revising processes of the manuscript. All authors read and approved the final manuscript.

## Funding

This research is supported by the National Natural Science Foundation of China (81671801, 61671300, 61472247), and the SJTU SMC-Morning Star Excellent Young Scholar-B (14X100010047).

### Conflict of interest statement

The authors declare that the research was conducted in the absence of any commercial or financial relationships that could be construed as a potential conflict of interest.
