# Supplementary material for: A 3D Computational Model of Transcutaneous Electrical Nerve Stimulation for Estimating Aβ Tactile Nerve Fiber Excitability
Source: Front Neurosci. 2017 May 16;11:250. doi: 10.3389/fnins.2017.00250 (PMC5432565; doi:10.3389/fnins.2017.00250)
Supplement: Supplementary file 1 [file DataSheet1.docx]

# Appendix

Dynamic equations for ionic channels were shown as follows. The equation parameters of activation and inactivation rates for the ions were derived from reported work (Howells et al., 2012). All the equation formations were based on the ion conductance referred from the double-cable-layer model.

Fast sodium

$$I_{Naf}=g_{Naf}*m^{3}*h*(V_{m}-E_{Na})$$

$$\alpha_{m}=\left[ 6.25*\left( V_{m}+18.3 \right) \right]/\left\{ 1-e^{[-\frac{V_{m}+18.3}{10.3}]} \right\}$$

$$\beta_{m}=\left\{ 0.289*\left[ -\left( V_{m}+22.6 \right) \right] \right\}/\left\{ 1-e^{[\frac{V_{m}+22.6}{9.16}]} \right\}$$

$$\alpha_{h}=\left\{ 0.153*\left[ -\left( V_{m}+113.8 \right) \right] \right\}/\left\{ 1-e^{[\frac{V_{m}+113.8}{11.9}]} \right\}$$

$$\beta_{h}=10.5/\left\{ 1+e^{[-\frac{V_{m}+31.6}{14.5}]} \right\}$$

Persistent sodium

$$I_{Nap}=0.0107*g_{Naf}*p^{3}*(V_{m}-E_{Na})$$

$$\alpha_{mp}=\left[ 3.13*\left( V_{m}+36.3 \right) \right]/\left\{ 1-e^{[-\frac{V_{m}+36.3}{10.3}]} \right\}$$

$$\beta_{mp}=\left\{ 0.145*\left[ -\left( V_{m}+40.6 \right) \right] \right\}/\left\{ 1-e^{[\frac{V_{m}+40.6}{9.16}]} \right\}$$

Fast potassium

$$I_{Kf}=g_{Kf}*n^{4}*(V_{m}-E_{K})$$

$$\alpha_{n}=\left[ 0.0221*\left( V_{m}+90.8 \right) \right]/\left\{ 1-e^{[-\frac{V_{m}+90.8}{7.7}]} \right\}$$

$$\beta_{n}=\left\{ 0.0393*\left[ -\left( V_{m}+73.6 \right) \right] \right\}/\left\{ 1-e^{[\frac{V_{m}+73.6}{7.35}]} \right\}$$

Slow potassium

$$I_{Ks}=g_{Ks}*s*(V_{m}-E_{K})$$

$$\alpha_{s}=\left[ 0.00563*\left( V_{m}+23.5 \right) \right]/\left\{ 1-e^{[-\frac{V_{m}+23.5}{12.7}]} \right\}$$

$$\beta_{s}=\left\{ 0.00341*\left[ -\left( V_{m}+91.1 \right) \right] \right\}/\left\{ 1-e^{[\frac{V_{m}+91.1}{11.7}]} \right\}$$
